# Socioeconomic determinants of low birth weight and its association with peripubertal obesity in Brazil

**DOI:** 10.3389/fpubh.2025.1424342

**Published:** 2025-03-19

**Authors:** Fernanda Lima-Soares, Renato Simões Gaspar, Silas Alves-Costa, Cecilia C. Costa Ribeiro, Antonio Marcus de Andrade Paes

**Affiliations:** ^1^Laboratory of Experimental Physiology, Department of Physiological Sciences, Biological and Health Sciences Centre, Federal University of Maranhão (UFMA), São Luís, Brazil; ^2^Health Sciences Graduate Program, Biological and Health Sciences Centre, Federal University of Maranhão (UFMA), São Luís, Brazil; ^3^Department of Pharmacology, Faculty of Medical Sciences, Universidade Estadual de Campinas, Campinas, Brazil; ^4^Graduate Program in Dentistry, Biological and Health Sciences Centre, Federal University of Maranhão (UFMA), São Luís, Brazil

**Keywords:** low birth weight, socioeconomic factors, peripubertal health, reproductive-age population, high body mass index, low- and middle income countries

## Abstract

**Introduction:**

Low birth weight (LBW) is an early life adversity associated with various risk factors and metabolic dysfunction throughout life. However, the role of socioeconomic factors in the association between LBW and peripubertal health in low- and middle-income countries (LMICs) remains unclear. This ecological study investigated the factors contributing to LBW and its impacts in Brazil.

**Methods:**

Data were collected from the Global Health Data Exchange as summary exposure values (SEVs), which serve as a proxy for population prevalence weighted by the relative risk. Additionally, information was sourced from official Brazilian government resources covering the years 1995 to 2017, resulting in a total of 338 state-year observations applied for temporal lagged analyses. First, we tested the SEV of 1-year lagged reproductive-age population (15–49 years) risk factors as exposures and the SEV of LBW as an outcome. In the second temporal lagged analysis, we tested the association between the SEV of LBW as the primary exposure and the SEV of high body mass index (HBMI) in peripubertal population 10 years later as the outcome. Fixed-effects multivariable linear regression models with lags were constructed, adjusting for socioeconomic covariates.

**Results:**

The exposure of the reproductive-age population to smoking, alcohol, high systolic blood pressure, and HBMI was positively associated with the SEV of LBW. A diet high in sugar-sweetened beverages (SSB diet) was also positively associated, but the association disappeared when GDP per capita and access to primary care were added to the model. Regarding the repercussions of LBW, a 1-point increase in the SEV of LBW was associated with a 1.6-point increase in HBMI in the peripubertal population (95% CI: 0.66 to 2.55). However, this association disappeared after adjusting for GDP per capita and access to primary care, indicating their confounding roles.

**Discussion:**

Our study highlights several risk factors in the adult population associated with LBW and its relationship with peripubertal HBMI. Interestingly, GDP per capita and access to primary care were found to be the socioeconomic determinants for birth outcomes as a result of exposure to the risk factors tested and the mid-term effects of LBW. These findings enhance our understanding of the role of socioeconomic factors contributing to LBW in LMICs and the need for public policies addressing healthcare and welfare to reduce the burden of LBW in LMICs.

## 1 Introduction

Low birth weight (LBW), whether due to preterm birth or fetal growth restriction, is one of the earliest outcomes of intrauterine malnutrition (1). It is defined as a birth weight lower than 2,500 g and is shown to increase the risk of death by over 20 times compared to normal-weight neonates (2). LBW is a well-known risk factor for several disorders, including overweight in both children and adults, type 2 diabetes, metabolic syndrome, and late cardiovascular disease (3, 4). The paradoxical condition of being born small, which leads to lasting effects of metabolic distress, has been studied since 1992, when Hales and Barker (5) established the thrifty phenotype concept after observing that birth weight influences the risk of type 2 diabetes and adiposity in adulthood. At a population level, another common paradoxical phenomenon is the double burden of malnutrition (DBM), i.e., the coexistence of undernutrition and overweight. This problem arises from the nutritional transitions exacerbating the demographic and epidemiological challenges in low- and middle-income countries (LMICs) (6).

Globally, one in four babies is born either prematurely or with low birth weight (1). In LMICs, intrauterine malnutrition is a prevalent issue, particularly due to the multifactor vulnerability to which the reproductive-age population is exposed (7). A joint report released in 2019 by the UNICEF and WHO indicated that between 2000 and 2015, an average of 75% of the world’s LBW neonates were born in LMICs (8). In addition to posing a risk of death, LBW serves as a valuable public health indicator of maternal health, nutrition, and poverty (9). An adverse maternal intrauterine environment influenced by factors such as malnutrition, neighborhood disadvantage, smoking, or emotional stress leads to early, medium, and long-term repercussions (2–4, 9, 10). These effects can be explained by adaptive developmental plasticity, a permanent physiological change in the developmental trajectory influenced by external or internal factors, occurring through gene–environment interactions (11).

Brazil is an LMIC and a large continental nation in Latin America, with unique characteristics making it an appropriate setting to investigate sociodemographic and prenatal risk factors associated with LBW. Data from the Surveillance System of Risk and Protective Factors for Chronic Diseases by Telephone Survey (Vigitel) indicate that overweight affects over 50% of the Brazilian population, while specific subgroups have shown increasing prevalence of underweight, characterizing an active DBM. This phenomenon appears to be influenced by social factors such as educational attainment, socioeconomic status, and demographic disparities (12). Similarly, data from the Cardiovascular Risk Factors in Adolescents Study (ERICA) indicate that these social factors are associated with an average 26.3% prevalence of excessive weight in adolescents aged 12 to 17 years in the country (13), reinforcing the idea that environmental and social factors can lead to metabolic disorders. Although the association between LBW and DBM has been hardly explored, it is reasonable to hypothesize the correlation between DBM and pubertal excessive weight in countries with a high prevalence of LBW.

In this regard, despite the consistent prevalence of LBW in Brazil, which nearly stands at 8.5% of all births (14), it remains unclear how the environmental and social factors influence the prevalence and the metabolic consequences of LBW. A broad understanding at the populational level of how modifiable factors lead to LBW and the subsequent repercussions of this condition on the health of future generations is essential for a thorough comprehension of its origins and for devising effective preventive strategies. Therefore, considering the life-long effects of LBW in general health, including late development of cardiometabolic diseases, and the scarcity of published data from LMICs, this study aimed to investigate the risk factors and socioeconomic covariates that potentially contribute to LBW occurrence and verify an outcome of this condition by determining the association between LBW and peripubertal excessive weight in Brazil.

## 2 Methods

### 2.1 Data source and study design

Data used in this ecological study were collected from publicly available datasets: the Global Health Data Exchange (GHDx), which is a comprehensive database on health-related variables also known as GBD study 2019 (15). Crude and age-standardized estimates of various measures of the burden of risk factors and LBW in Brazilian States from 1995 to 2017 were extracted from the GBD database^1^ with no specific permissions required. In addition, official sources from the Brazilian government were used to collect data related to socioeconomic covariates, namely, poverty data that were collected from the Brazilian Ministry of Social Development, access to primary care and hospital support from the Brazilian Ministry of Health, and the Gini index and GDP per capita data from the Brazilian Institute of Geography and Statistics (IBGE).

GBD estimates incidence, prevalence, summary exposure value (SEV), mortality, years of life lost (YLLs), years lived with disability (YLDs), and disability-adjusted life-years (DALYs) due to 369 diseases and injuries, for both sexes, and for 204 countries and territories. Input data were extracted from censuses, household surveys, civil registration and vital statistics, disease registries, health service use, air pollution monitors, satellite imaging, disease notifications, and other sources (15). Among these estimations, SEV was used to compare the distribution of excess risk multiplied by exposure level to a population where everyone is at maximum risk. SEV represents a prevalence-weighted measure of the relative risk associated with exposure to various disease-causing risk factors. The relative risk, derived from both primary research (published and unpublished) and secondary review studies, quantifies the likelihood of developing a disease or experiencing mortality or morbidity due to specific risk factor exposures. SEVs range from 0 to 100%, where 0 denotes the absence of excess risk in the population and 100% corresponds to the maximum level of exposure risk. An increasing SEV reflects a higher prevalence of the risk factor within the population, whereas a decreasing value indicates a reduction in its prevalence (16). Multivariable linear regression models were constructed from those datasets, as described in Section 2.4.

### 2.2 Risk factors

The prevalence of modifiable risk factors was computed as a summary exposure value (SEV) in GHDx 2019 (17), which denotes the prevalence weighted by the relative risk, demonstrating the extent of exposure by the risk level and the severity of that risk contribution to disease burden. Risk factors included are tobacco use (smoking), alcohol use (alcohol), low physical activity (LPA), diet high in trans fatty acids (TFA diet), diet high in sugar-sweetened beverages (SSB diet), increased levels of plasma glucose (glucose), high systolic blood pressure (HSBP), diet high in LDL cholesterol (HLDL), and high body mass index (HBMI). All data were age-standardized and stratified by sex.

### 2.3 Covariates

As previously described (18), we included the socioeconomic determinants and access to healthcare covariates in both models to bring the model closer to the social complexity in which individuals are included. The selected covariates were Gini index of household income, the main metric used for determining income inequality; gross domestic product (GDP) per capita in R$ (Brazilian Reais), an income variable also capable of being associated with the population health (19); number of hospital beds per 1,000 inhabitants; and coverage of primary care (20) to account for access to healthcare. To assess poverty, Bolsa familia value in R$ (Reais) per 1,000 individuals, a Brazilian state income transfer program for low-income families, was used (21). The covariates collected ranged from 2004 to 2016. To account for heteroskedasticity and autocorrelation, which are common when dealing with panel data, a correlation panel was created for each model including covariates and variables (Supplementary Tables 1, 2).

### 2.4 Statistical analysis

**Figure 1:**
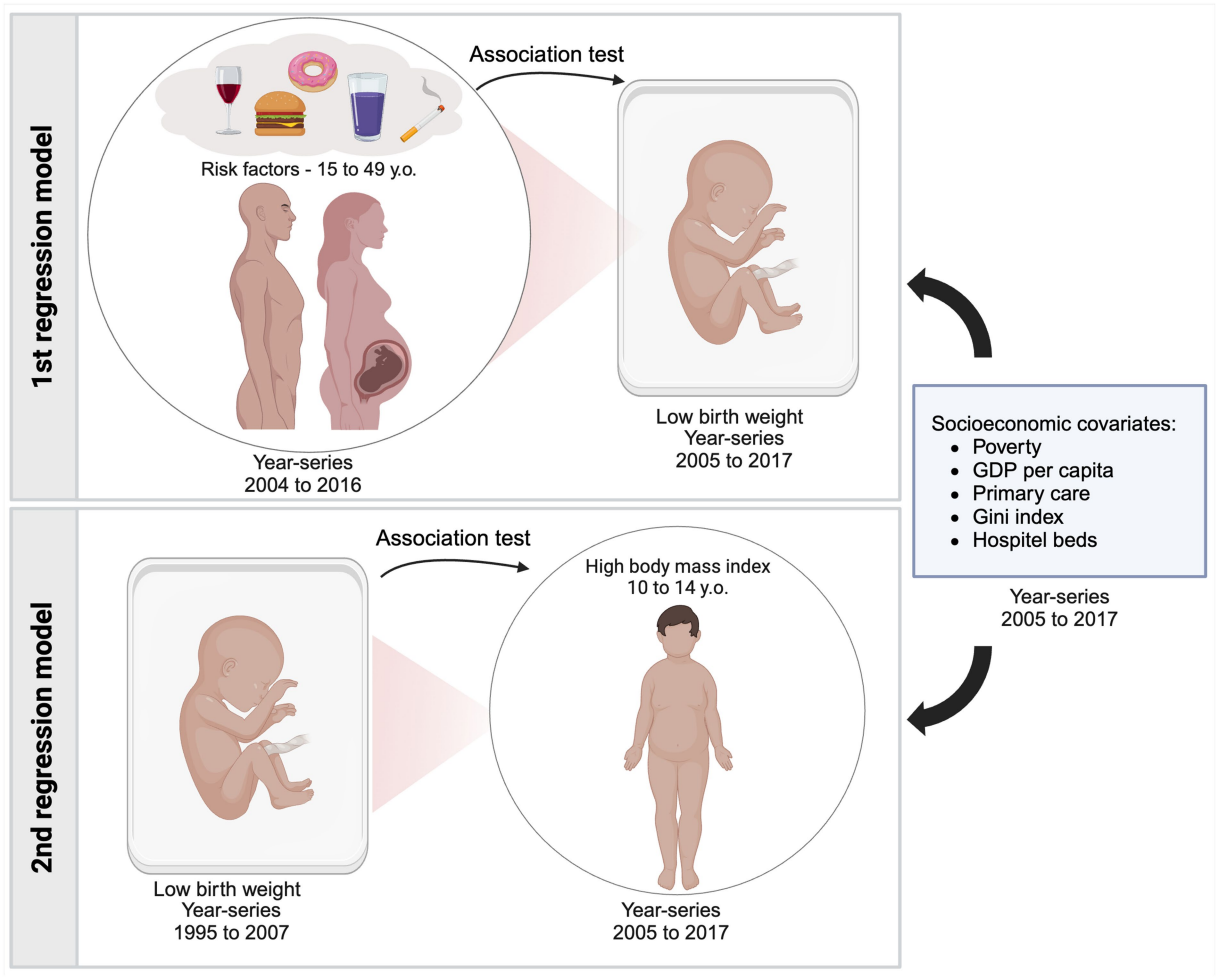
Study design: presentation of different year-series populations included in each model and the socioeconomic covariates used as adjustments. In the first regression model 1, SEV of several risk factors from male and female populations aged 15 to 49 years was tested in association with SEV of low birth weight 1 year later. The second regression model tested the association between SEV of low birth weight and high body mass index in populations aged10 to 14 years, 10 years later. Created with BioRender.com.

Multivariable linear regression models including data from all 26 Brazilian States, henceforth referred to as States, in different year-series totaling 338 State-year observations to each variable were performed using R software (22). Figure 1 summarizes the whole study population, data year-series, and covariates. Different models were constructed. First, a 1-year temporal lag model was utilized to assess the associations between reproductive-age population risk factors (2004–2016), where each risk factor was used as an independent variable (predictor), and LBW (2005–2017), as a dependent variable. Then, a 10-year lag model was applied to evaluate the association between LBW (1995–2007) as an independent variable (predictor) and peripubertal high body mass index (HBMI) (2005–2017) as a dependent variable (outcome). Data were stratified by sex. Reproductive-age population was defined as men and women aged between 15 and 49 years (17), while peripubertal population was defined as boys and girls aged 10 to 14 years. For all variables and covariates in the selected time series, the retrieved dataset was complete, with no missing data. While risk factors and LBW were expressed as SEVs, other aforementioned covariates were expressed as prevalence and assumed as potential confounders.

As a standard procedure for data processing, the analysis began with testing the crude association between the predictor and the outcome, without adjustment for covariates. If a positive association was observed, a new model was created by including all adjustment covariates. In cases where this adjustment consistently blocked the association, a stepwise adjustment procedure was applied, where covariates were added individually to the model. This stepwise approach allowed for identifying specific covariates responsible for blocking the association. In the context of multivariable linear regression analysis, this method facilitates in determining the covariate primarily influencing changes in the association. By isolating this key covariate, it is possible to identify its role in altering the outcomes and analyze the interplay between different exposures and their effects on the results (23).

State-fixed effects were employed to account for intrinsic cultural, geographical, and historical characteristics that may vary across States but are fixed over time. The fixed-effects model is a robust and conservative analytical instrument well suited for examining aggregated health data (18) and was estimated using ordinary least square (OLS), which is employed to predict unknown parameters in a linear regression model. The use of the fixed-effects model aided in reducing the likelihood of multiplicity in the model. The output of the model can be interpreted as changes in the dependent variable associated with a 1-point change in the SEV for a given risk factor over time and is presented as the estimated coefficients and their associated 95% confidence interval (2.5 and 97.5% bounds).

## 3 Results

### 3.1 Association between reproductive-age population risk factors and LBW

To assess whether the exposure of male and female reproductive-age populations to several risk factors would be associated with SEV of LBW of newborn boys and girls 1 year after the exposure, a 1-year temporal lag model was created and applied. A summary of the State-level data on risk factors, socioeconomic determinants, and SEV of LBW is presented in Supplementary Tables 3, 4. As shown in Table 1, multivariable linear regression analyses showed a direct positive association of alcohol, tobacco, and SSB diet with SEV of LBW for both sexes. HBMI is also positively associated with SEV of LBW, except for HBMI in men and SEV of LBW in boys. Regarding HSBP, reproductive-age women showed a significant association with SEV of LBW for both girls and boys, while for men, HSBP was associated with SEV of LBW in boys but not girls (Table 1).

**Table 1:** Multivariable linear regression from several risk factors of the male and female reproductive-age population with SEV of LBW in newborn boys and girls.

Reproductive-age risk factors	Newborn girls		Newborn boys	
	Coefficient (95% CI)	*p*-value	Coefficient (95% CI)	*p*
Women				
Alcohol	0.71 (0.23; 1.19)	**0.003**	0.92 (0.48; 1.37)	**0.0001**
SSB diet	0.08 (0.02; 0.14)	**0.007**	0.11 (0.06; 0.16)	**0.0001**
TFA diet	0.05 (−0.42; 0.14)	0.281	0.04 (−0.05; 0.13)	0.395
HBMI	0.15 (0.04; 0.27)	**0.008**	0.18 (0.10; 0.27)	**0.0001**
Glucose	−0.35 (−1.4; 0.69)	0.508	−0.35 (−1.2; 0.49)	0.411
HLDL	−0.02 (−0.40; 0.34)	0.883	0.10 (−0.31; 0.52)	0.618
HSBP	0.23 (0.11; 0.35)	**0.0001**	0.21 (0.10; 0.33)	**0.0001**
LPA	−0.07 (−0.38; 0.23)	0.62	−0.26 (−0.59; 0.06)	0.11
Smoking	0.31 (0.18; 0.43)	**0.0001**	0.32 (0.19; 0.45)	**0.0001**
Men				
Alcohol	0.48 (0.09; 0.86)	**0.01**	0.65 (0.28; 1.03)	**0.0005**
SSB diet	0.1 (0.02; 0.19)	**0.01**	0.14 (0.07; 0.21)	**0.0001**
TFA diet	0.06 (−0.01; 0.14)	0.092	0.07 (0,0; 0.15)	0.057
HBMI	0.11 (−0.01; 0.25)	0.07	0.15 (0.04; 0.27)	**0.005**
Glucose	−0.05 (−1.0; 0.9)	0.918	−0.05 (−0.72; 0.71)	0.988
HLDL	−0.06 (−0.36; 0.35)	0.97	0.14 (−0.22; 0.51)	0,448
HSBP	0.12 (0.00; 0.24)	0.051	0.11 (0.05; 0.23)	**0.03**
LPA	0.01 (−0.23; 0.26)	0.91	−0.07 (−0.35; 0.2)	0.50
Smoking	0.32 (0.15; 0.49)	**0.0002**	0.37 (0.23; 0.52)	**0.0001**

Bold values correspond to statistically significant *p*-values (*p* ≤ 0.05).

**Figure 2:**
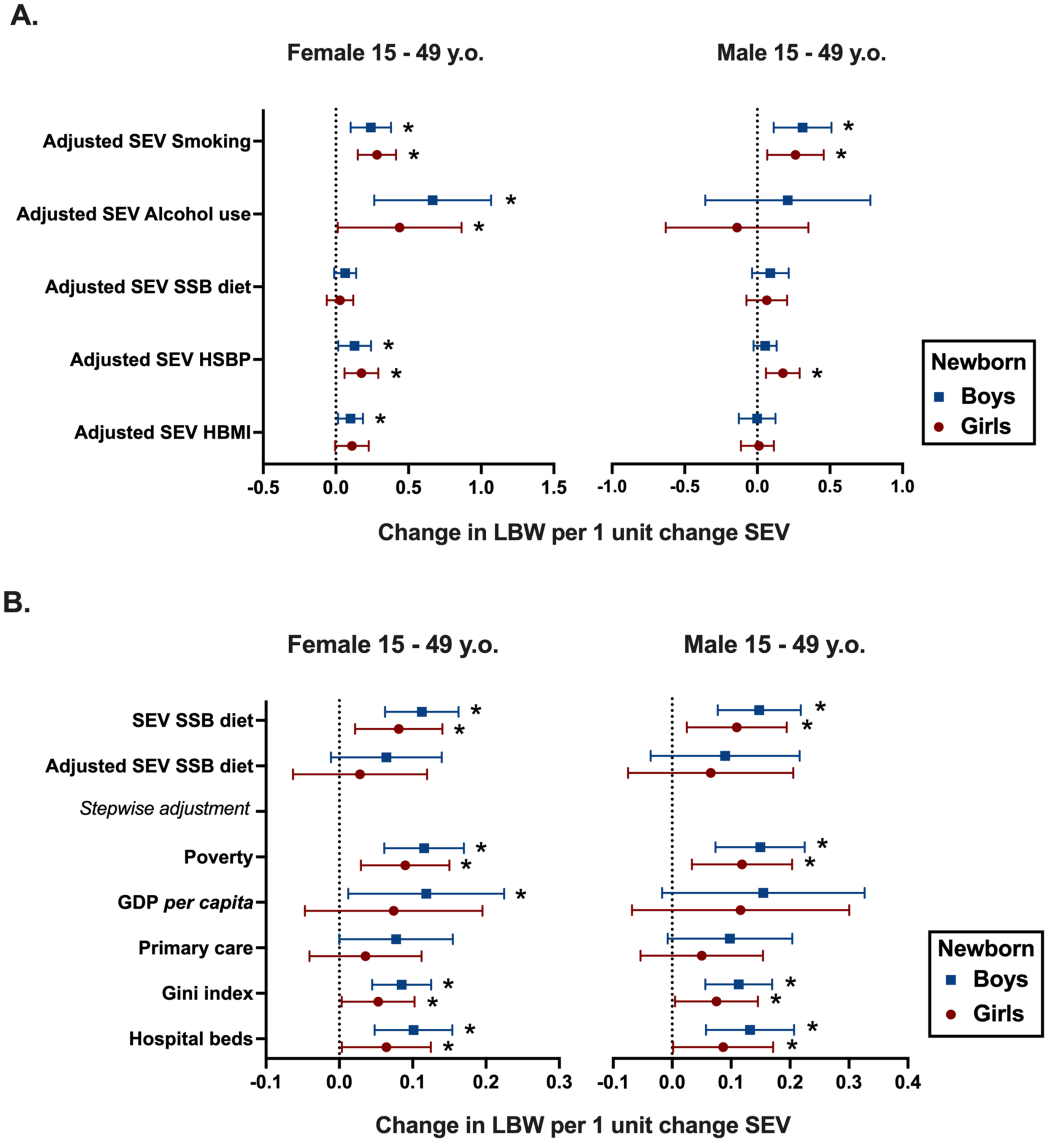
Risk factors of the female and male reproductive-age population (15–49 years old) associated with LBW in Brazilian States. **(A)** Multivariable linear regression analysis shows the risk factors of the male and female reproductive-age population associated with SEV of LBW in newborn boys and girls when fully adjusted for socioeconomic covariates. **(B)** Fully adjusted and Unadjusted regression models of SEV SBB diet with SEV LBW, followed by Stepwise adjustment from female and male population. Socioeconomic covariates: Gini index of household income; gross domestic product (GDP) per capita in R$ (Brazilian Reais); number of hospitals beds per 1,000 inhabitants; coverage of primary care; and Log Bolsa familia as poverty. The variables State- and year-fixed effects are included in the model. Risk factors are exhibited as follows: smoking as tobacco use, alcohol use, HSBP as high systolic blood pressure, SSB diet as diet high in sugar-sweetened beverages, and HBMI as high body mass index. * *p* < 0.05.

To enhance the robustness and accuracy of the analyses, the effects of socioeconomic covariates, Gini index, GDP per capita, hospital beds per 1,000 inhabitants, coverage of primary care, and poverty were incorporated as adjustments to the model, corresponding to the risk factors already identified as associated with LBW in Table 1. With such adjustments, as shown in Figure 2A, SEV for smoking was the only risk factor of the male population that sustained the association with LBW in both girls (0.26; 95% CI 0.06 to 0.4) and boys (0.31; 95% CI 0.1 to 0.5). In addition to smoking, SEV of HSBP in men was also associated with LBW, but only in girls (0.07; 95% CI 0.007 to 0.14). For the female population, several risk factors preserved the positive association with LBW for both girls and boys, which include SEVs of smoking (girls 0.28; 95% CI 0.1 to 0.4 and boys 0.24; 95% CI 0.1 to 0.37), alcohol (girls 0.43; 95% CI 0.01 to 0.8 and boys 0.6; 95%CI 0.26 to 1.00), and HSBP (girls 0.02; 95% CI 0.05 to 0.11 and boys 0.12; 95% CI 0.01 to 0.24). However, SEV of HBMI in women was associated with LBW only in boys (0.10; 95% CI 0.01 to 0.18) (Figure 2A).

High SSB diet was the only positive association to be lost after full adjustment with covariates, representing a consistent response for both male and female reproductive-age populations despite the newborn sex (Figure 2A). Therefore, we performed further analyses employing stepwise addition of individual socioeconomic determinants, allowing the identification of potential covariates that may be confounding the association. Figure 2B shows that the SEV for the number of hospital beds, Gini index, and poverty did not change the positive association between SSB diet and LBW. On the other hand, stepwise adjustment for SEV coverage of primary care for men (girls 0.05; 95% IC −0.05 to 0.1 and boys 0.09; 95% IC −0.007 to 0.2) and women (girls 0.03; 95% IC −0.04 to 0.1 and boys 0.07; 95% IC −0.001 to 0.1) at reproductive age avoided the association with LBW in newborns from both sexes. Similarly, adjustment for SEV of GDP per capita blocked the association of SEV of SSB diet in men with LBW in newborn boys (0.1; 95% IC −0.01 to 032) and girls (0.1; 95% IC −0.06 to 0.3), while in women, it only prevented the association with LBW in girls (0.07; 95% IC −0.041 to 0.1) (Figure 2B).

Altogether, our data regarding socioeconomic and modifiable risk factors among male and female reproductive-age populations leading to LBW 1 year later supporting a sexually dimorphic association, once variables in women, such as HBMI and HSBP, appear to be more steadily associated with LBW in women than in men. Moreover, the exposure to SSB diet *per se* is associated with LBW, albeit the improvement of primary care or GDP per capita is possibly able to nullify it.

### 3.2 Association between LBW and peripubertal high body mass index

**Figure 3:**
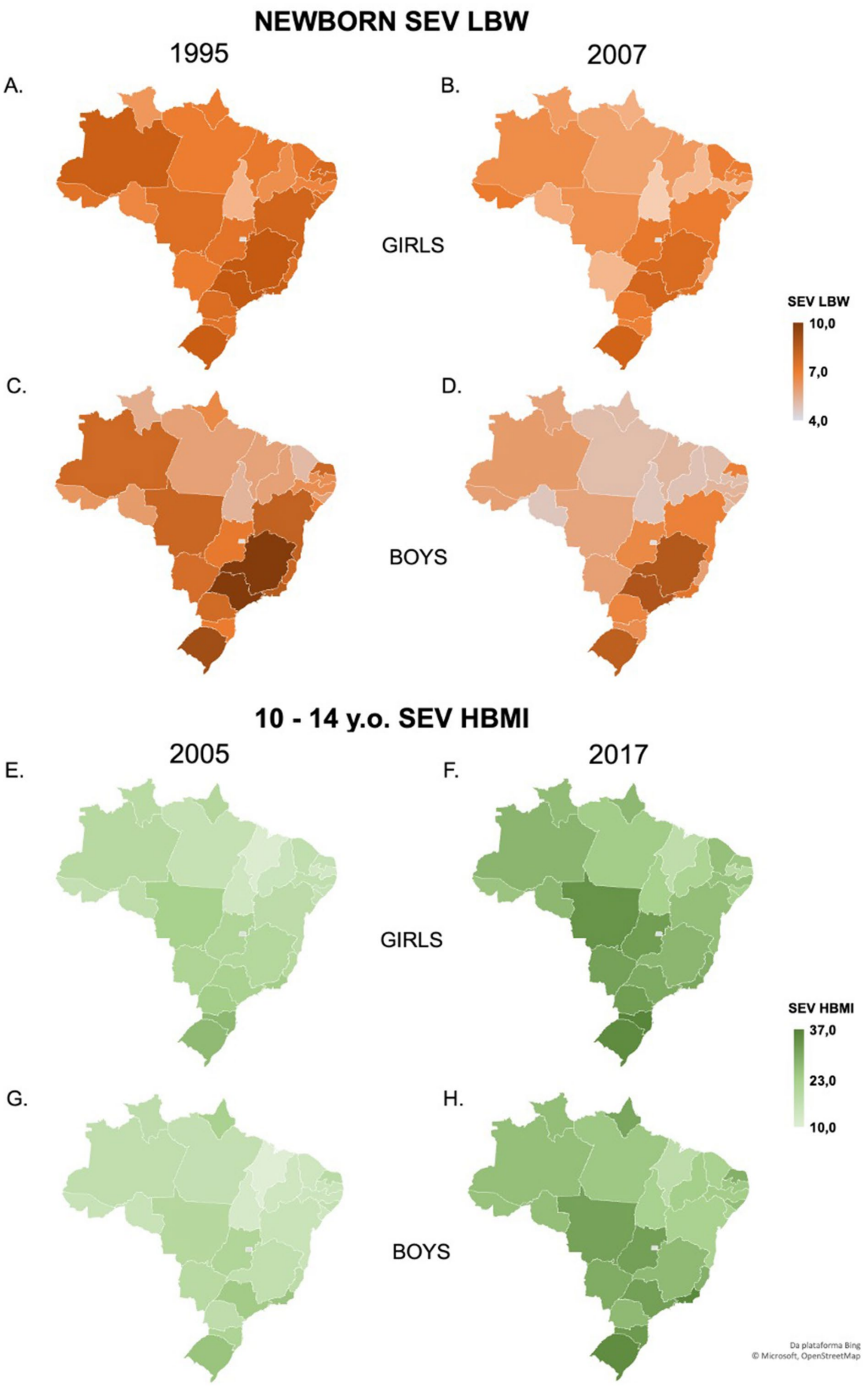
Summary exposure value (SEV) distribution of male and female SEV of LBW and peripubertal SEV of HBMI in Brazilian States in the 10-year lagged model. The maps display the SEV of LBW from 1995 **(A)** to 2007 **(B)** for girls, and 1995 **(C)** to 2007 **(D)** for boys. SEV of HBMI 10–14 years old from 2005 **(E)** to 2017 **(F)** for girls and 2005 **(G)** to 2017 **(H)** for boys. LBW: low birth weight; HBMI: high body mass index. Data were obtained from the Global Health Data Exchange (GHDx).

After analyzing the risk factors associated with LBW, we next proceeded to investigate how/whether LBW *per se* is associated with HBMI in the peripubertal age population. Thus, the second model data from SEV of LBW in newborn girls and boys and SEV of HBMI in peripubertal girls and boys using the same time series were applied in the multilinear models as shown in Figure 3. Between 1995 and 2007, the countrywide average SEV of LBW decreased by 17% in female (7.8 to 6.6, Figures 3A,B) and 15% in male (7.18 to 6.11, Figures 3C,D) neonates. In the meantime, the 10-year lagged (2005 to 2017) countrywide average SEV of HBMI in adolescents aged 10 to 14 years increased by 43% in girls (19 to 27.1, Figures 3E,F) and 52% in boys (17.7 to 27, Figures 3G,H), depicting an inversely proportional behavior of those variables over time in Brazil.

**Figure 4:**
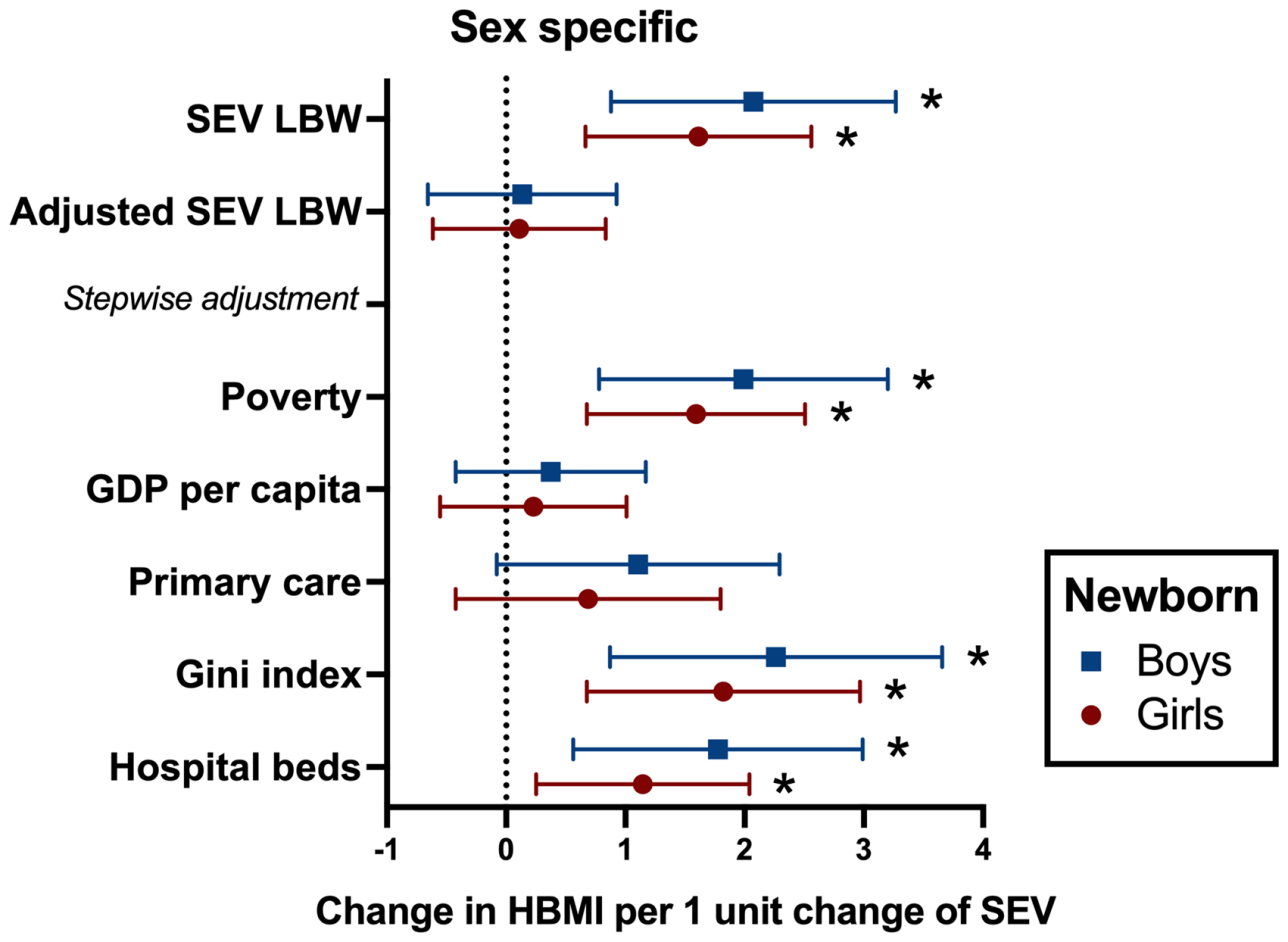
Association between SEV of LBW and peripubertal HBMI in the 10-year lagged model with stepwise addition of covariates. The variables State- and year-fixed effects are included in the model. Data are presented as estimate and 95% confidence intervals. LBW: low birth weight. HBMI 10–14 yo: high body mass index between 10 and 14 years old. Covariates: poverty as Bolsa familia value in R$ (Reais) per 1,000 individuals; GDP per capita as gross domestic product (GDP) per capita in R$ (Brazilian Reais); coverage of primary care per 1,000 inhabitants; Gini index; and number of hospital beds per 1,000 inhabitants.

To test the State-fixed association between LBW and pubertal HBMI, crude and multivariable linear regressions were performed adjusting for covariates models, as previously described. When unadjusted, each 1-point increase in SEV of LBW was associated with a consistent increase of peripubertal SEV of HBMI in girls (1.6; 95% CI 0.66 to 2.55) and boys (2; 95% CI 0.8 to 3.2) (Figure 4). However, in multivariable analysis including all covariates, such association was lost, regardless of sex. To recognize the socioeconomic covariates that exerted a greater influence in the adjustment, the stepwise addition of covariates was performed. The individual adjustment for poverty, hospital beds, or Gini index did not block the association between LBW and HBMI between 10 and 14 years. Meanwhile, after adjusting for GDP per capita, both girls (0.2; 95% CI −0.5 to 1) and boys with LBW (0.3; 95% CI −0.4 to 1.1) were no longer associated with peripubertal HBMI. A similar result was obtained after adjusting for the coverage of primary care for both girls (0.6; 95% CI −0.4 to 1.7) and boys (1.1; 95% CI −0.07 to 2.2), suggesting that GDP per capita and primary care appear to be confounders to the burden of peripubertal HBMI among girls and boys born small (Figure 4).

## 4 Discussion

The current study has shed light on the role of income per capita and access to primary care regarding the LBW rates in Brazil. Moreover, these social determinants also acted in the double burden of malnutrition, influencing LBW and peripubertal HBMI 10 years later. This main result is supported by the evidence that the association between the exposure of reproductive-age population to an SSB-rich diet and the occurrence of LBW was decreased in models including GDP per capita or coverage of primary care, which also prevented the association between LBW and HBMI 10 years later. It is noteworthy that these covariates blocked the association between the exposure of reproductive-age men and LBW in neonates from both sexes, highlighting the negative impact of paternal exposure on fetal health outcomes. Overall, the findings herein presented emphasize the need for public health policies addressing the reproductive-age population from both sexes to prevent LBW and its repercussions for future generations, especially in LMICs.

The detrimental effects of maternal smoking, alcohol use, HSBP, and HBMI prior to or during pregnancy on fetal health have been well documented, with evidence demonstrating a higher risk of preterm birth and LBW in several populations (24–33). On the other hand, emerging data have also highlighted the role of paternal preconception exposure to diverse risk factors negatively impacting fetal growth and lifelong health outcomes in the offspring (34–36). Therefore, considering both male and female exposure, we first created models directly associating female and male reproductive-age population risk factors with LBW, without adjustments. Our findings based on Brazilian States data align with those of previous studies. They supported a positive association of both male and female exposure to smoking, alcohol use, and HSBP with SEV LBW 1 year later, corroborating the association between LBW and male exposure to such risk factors.

Regarding HBMI in reproductive-age population, our results showed that pre-existent excess weight in women is positively associated with LBW, corroborating with previous studies from Korean, Polish, and Danish populations which showed that maternal preconceptional obesity increased the risk of LBW (24, 37, 38). When associating HBMI in men with LBW, we found a positive association with newborn boys, but not for newborn girls, suggesting a sex-specific role for paternal HBMI on LBW. Noteworthily, some studies have found a positive association between postconceptional obesity and maternal gain during gestation and a higher risk of the offspring being born large for gestational age or macrosomic (39, 40). Such data support distinct roles for the impact of maternal obesity on birth weight, depending on the time it occurs. Meanwhile, data concerning the impact of paternal obesity on such outcomes are still elusive despite the current debate about the paternal origins of health and disease (POHaD) (35, 36).

Transgenerational inheritance depends on genetic, epigenetic, and environmental aspects, varying in different populations, socioeconomic scenarios, and the aforementioned risk factor exposures (41). Thus, to bring our model closer to a real environment, socioeconomic adjustments at State-level data were added. In accordance with the historical association of maternal smoking or alcohol use with LBW in several populations (25, 30, 41–44), our data sustained this positive association even after adjustments were added. However, for alcohol use in men, the association with LBW was lost when socioeconomic data were added. Notwithstanding, when adjusted, female HSBP remained associated with LBW, corroborating the recent findings by Ishikuro et al. (32) in the Japanese population. Despite the inherent limitations of our population-based dataset, which precluded us from directly linking parents and offspring within individual-level associations, it does allow us to speculate that the same health- and lifestyle-associated risk factors apply for the understanding of preconceptional etiology of LBW and their role as transgenerational risk factors for non-communicable chronic diseases.

Considering malnutrition as one of the fundamental aspects of the environment, it is crucial to not reduce it to calorie intake, since it includes the assessment of macro- and micronutrient intake, as well as healthy or unhealthy food consumption, as classified by the Prime Diet Quality Score (PDQS) (28, 45). Unhealthy food includes SSB diet, and their inadequate consumption during pregnancy can impair fetal growth and development (28, 46). In a prospective study on Norwegian women, Englund-Ögge et al. (47) demonstrated that the lower the maternal education level, the higher is the positive association with maternal SSB intake, maternal HBMI, and preterm birth. Similarly, our unadjusted model showed that exposure of men and women to a diet high in SSB was positively associated with LBW, even though this association was lost when all the socioeconomic covariates were added. On the other hand, stepwise adjustments identified GDP per capita and access to primary care as the covariates responsible for preventing the association between a diet high in SSB and LBW in the Brazilian States, strengthening the evidence that socioeconomic status can modulate the impact of SSB ingestion on gestational outcomes.

Social disadvantage, especially low income, has been associated with intrauterine impairment of brain development, while increasing the access to social welfare programs as a way to reduce the burden of social disadvantage has the capacity to detain such outcomes (10, 48). This finding aligns with our results since access to primary care and GDP per capita played a pivotal role in the association of the exposure of reproductive-age population to SSB with LBW, highlighting the relevance of socioeconomic status and healthcare for perinatal health. Although the data presented herein are not sufficient to assert the overall protective effect of increased access to primary care and higher income per capita against LBW occurrence, they consistently suggest that in a context of high SSB intake, they may protect the fetus from being born small, a major consequence of intrauterine malnutrition, with a positive impact on its lifelong health status.

When investigating the mid-term repercussions of being born small, there was a positive association between LBW and HBMI in peripubertal girls and boys 10 years later, which was lost if income per capita and access to primary care were added to the model. This finding is corroborated by the concept of the developmental origins of health and disease (DOHaD), which has long hypothesized and examined the biological relationship between LBW and body weight gain throughout the lifespan (49, 50). Numerous studies have implicated LBW as a risk factor for obesity development in different populational groups (4, 49, 51–53). However, this study puts a spotlight on the unique role of primary care and GDP per capita in attenuating the association between being born with LBW and increasingly gaining weight over time. According to our data, Brazilian States with better access to primary care and higher income per capita were able to mitigate the mid-late metabolic effects of low birth weight, which supports the increasing notion that LBW is not a deterministic condition in terms of health or disease but rather a risk factor. LBW may lead to either a negative or non-negative outcome, depending on genetic, environmental, and socioeconomic status, as we have presented (4, 54–56).

Brazil is an LMIC historically marked by poverty and income inequality, with above 50% of the population facing some level of food insecurity (57), which helps explain the consistent rates of LBW over the years (~8.5%) (14). However, there are scarce data on the health status of those LBW newborns throughout their life cycle. Having said that, studies including long-term follow-up of children born small to assess the lasting effects of this condition in different scenarios are needed, as well as interventional research aiming to circumvent the metabolic disability. In addition to the limitations of our study, including that analyzed data were from a few years ago, because of lacking some recent year-series information in Brazilian sources, the study’s findings advocate for comprehensive public health interventions aimed at preventing the reproductive-age population exposure to well-known risk factors causing intrauterine malnutrition. Nonetheless, our data highlight the intricate interplay of socioeconomic factors and healthcare in influencing birth outcomes and call for targeted strategies to enhance male and female healthcare, ultimately fostering healthier outcomes for both mothers and their offspring.

Overall, this study provides evidence from an LMIC, Brazil, where alcohol consumption, smoking, high systolic blood pressure, high body mass index, and diet high in sugar-sweetened beverages in the population between 15 and 49 years old were associated with LBW in a State level. In addition, LBW is associated with a high body mass index of children between 10 and 14 years old. Remarkably, in both models, income and primary care played a pivotal role, suggesting that rich societies with high access to healthcare were favored to overcome the harmful effects of LBW on peripubertal metabolic health.

## Data Availability

The original contributions presented in the study are included in the article/Supplementary material, further inquiries can be directed to the corresponding author.

## Footnotes

^1^ http://ghdx.healthdata.org/gbd-results-tool
