# Supplementary material for: Socioeconomic determinants of low birth weight and its association with peripubertal obesity in Brazil
Source: Front Public Health. 2025 Mar 19;13:1424342. doi: 10.3389/fpubh.2025.1424342 (PMC11961654; doi:10.3389/fpubh.2025.1424342)
Supplement: Supplementary file 1 [file Table_1.docx]

**Supplementary Table 1**. Multiple correlation between socioeconomic covariates and risk factors

|  | CPC | GDP | Gini¹ | Poverty² | NHB³ | Alcohol | SSB diet | TFA diet | HBMI | Glucose | HLDL | HSBP | LPA | Tobacco |
| --- | --- | --- | --- | --- | --- | --- | --- | --- | --- | --- | --- | --- | --- | --- |
| CPC | 1 |  |  |  |  |  |  |  |  |  |  |  |  |  |
| GDP² | -0,251 | 1 |  |  |  |  |  |  |  |  |  |  |  |  |
| Gini¹ | -0.362 | 0.152 | 1 |  |  |  |  |  |  |  |  |  |  |  |
| Poverty³ | 0.271 | 0.109 | -0.029 | 1 |  |  |  |  |  |  |  |  |  |  |
| NHB¹ | -0.308 | -0.146 | 0.165 | -0.176 | 1 |  |  |  |  |  |  |  |  |  |
| Alcohol | -0,323 | 0,564 | 0,154 | 0,003 | 0,405 | 1 |  |  |  |  |  |  |  |  |
| SSB diet | -0,587 | **0,830** | 0,295 | -0,193 | 0,250 | 0,575 | 1 |  |  |  |  |  |  |  |
| TFA diet | -0,259 | -0,562 | 0,028 | -0,474 | 0,311 | -0,063 | -0,190 | 1 |  |  |  |  |  |  |
| HBMI | -0,278 | **0,828** | 0,075 | 0,162 | 0,009 | 0,438 | **0,721** | -0,661 | 1 |  |  |  |  |  |
| Glucose | 0,289 | 0,266 | -0,253 | 0,476 | 0,011 | 0,109 | -0,046 | -0,537 | 0,298 | 1 |  |  |  |  |
| HLDL | 0,184 | 0,392 | -0,187 | 0,006 | -0,191 | 0,237 | 0,228 | -0,418 | 0,493 | 0,090 | 1 |  |  |  |
| HSBP | -0,327 | 0,509 | 0,242 | 0,184 | 0,485 | 0,752 | 0,505 | 0,000 | 0,376 | 0,143 | 0,032 | 1 |  |  |
| LPA | 0,076 | -0,053 | 0,038 | -0,071 | 0,005 | -0,552 | -0,079 | -0,079 | -0,003 | -0,004 | -0,133 | -0,273 | 1 |  |
| Smoking | -0,543 | 0,217 | 0,206 | -0,408 | 0,606 | 0,479 | 0,552 | 0,410 | 0,183 | -0,305 | -0,020 | 0,514 | -0,178 | 1 |

¹per 1,000 inhabitants; ²per capita in R$ (Brazilian Reais); ³Bolsa familia;

**Supplementary Table 2**. Multiple correlation between socioeconomic covariates and LBW

|  | CPC | GDP | Gini index¹ | Poverty² | NHB³ | LBW |
| --- | --- | --- | --- | --- | --- | --- |
| CPC¹ | 1 |  |  |  |  |  |
| GDP² | -0,251 | 1 |  |  |  |  |
| Gini index² | -0.362 | 0.152 | 1 |  |  |  |
| Poverty³ | 0.271 | 0.109 | -0.029 | 1 |  |  |
| NHB¹ | -0.308 | -0.146 | 0.165 | -0.176 | 1 |  |
| LBW | -0.540 | 0.117 | 0.610 | -0.178 | 0.442 | 1 |

¹per 1,000 inhabitants; ²per capita in R$ (Brazilian Reais); ³Bolsa familia;

**Supplementary Table 3.** Summary of male and Female SEV risk factor variables among 2004 and 2016 from all 26 States.

|  | 15 to 49 y.o. risk factors (2004 – 2016) | | | | | | | | | | | | | | | | | | | | | | | | | | |
| --- | --- | --- | --- | --- | --- | --- | --- | --- | --- | --- | --- | --- | --- | --- | --- | --- | --- | --- | --- | --- | --- | --- | --- | --- | --- | --- | --- |
|  | SEV Alcohol use | | | SEV Diet high in Sugar sweetened beverages | | | SEV Diet high in Trans fatty acids | | | SEV High body mass index | | | SEV High fasting plasma glucose | | | SEV High LDL cholesterol | | | SEV High systolic blood pressure | | | SEV Low physical activity | | | SEV Tobacco use | | |
| *State* | Mean | min | máx | Mean | min | max | Mean | min | máx | Mean | min | máx | Mean | min | máx | Mean | min | máx | Mean | min | máx | Mean | min | máx | Mean | min | máx |
| Acre | 8,63 | 8,56 | 8,72 | 41,64 | 41,05 | 42,66 | 45,81 | 43,70 | 50,11 | 25,00 | 19,20 | 29,63 | 3,86 | 3,54 | 4,16 | 37,89 | 36,53 | 39,25 | 11,83 | 11,57 | 12,39 | 13,20 | 12,95 | 13,48 | 18,53 | 16,76 | 20,73 |
| Alagoas | 9,51 | 9,38 | 9,59 | 37,79 | 37,27 | 38,86 | 45,83 | 43,72 | 50,07 | 22,24 | 17,07 | 26,52 | 5,19 | 4,62 | 5,62 | 37,00 | 35,92 | 38,16 | 13,29 | 13,00 | 13,90 | 12,23 | 12,01 | 12,55 | 16,87 | 15,96 | 17,94 |
| Amapá | 10,26 | 10,09 | 10,38 | 42,96 | 42,39 | 43,95 | 45,82 | 43,74 | 50,07 | 27,80 | 23,13 | 31,72 | 3,82 | 3,44 | 4,19 | 39,13 | 38,09 | 40,42 | 10,97 | 10,69 | 11,58 | 12,48 | 12,32 | 12,67 | 15,77 | 15,12 | 16,70 |
| Amazonas | 8,84 | 8,73 | 8,94 | 46,77 | 46,15 | 47,60 | 45,87 | 43,75 | 50,09 | 25,52 | 20,70 | 29,77 | 3,80 | 3,41 | 4,16 | 38,51 | 37,43 | 39,83 | 9,74 | 9,53 | 10,28 | 13,48 | 13,29 | 13,69 | 15,80 | 14,62 | 17,07 |
| Bahia | 10,72 | 10,59 | 10,79 | 39,91 | 39,36 | 40,94 | 45,81 | 43,70 | 50,10 | 22,86 | 17,75 | 26,98 | 4,42 | 3,92 | 4,75 | 37,71 | 36,66 | 38,78 | 15,67 | 15,34 | 16,36 | 11,55 | 11,38 | 11,72 | 15,66 | 14,45 | 16,96 |
| Ceará | 9,63 | 9,54 | 9,69 | 37,95 | 37,28 | 39,34 | 45,90 | 43,80 | 50,05 | 23,88 | 18,47 | 28,15 | 4,03 | 3,62 | 4,28 | 38,07 | 36,87 | 39,15 | 14,47 | 14,16 | 14,96 | 14,71 | 14,02 | 14,97 | 16,77 | 15,41 | 18,62 |
| Espírito Santo | 10,90 | 10,76 | 11,03 | 50,44 | 49,04 | 53,05 | 45,88 | 43,88 | 50,07 | 25,77 | 20,29 | 30,62 | 4,47 | 4,23 | 4,63 | 39,58 | 38,42 | 40,80 | 13,96 | 13,75 | 14,47 | 12,22 | 12,01 | 12,49 | 18,19 | 16,34 | 20,41 |
| Goiás | 10,55 | 10,42 | 10,72 | 44,80 | 43,85 | 46,69 | 45,91 | 43,84 | 50,04 | 25,30 | 20,05 | 29,65 | 4,15 | 3,76 | 4,48 | 38,78 | 37,58 | 40,05 | 14,70 | 14,40 | 15,24 | 11,43 | 11,20 | 11,70 | 19,56 | 17,86 | 21,60 |
| Maranhão | 9,13 | 8,92 | 9,31 | 36,19 | 35,78 | 37,22 | 45,92 | 43,82 | 50,10 | 17,81 | 12,93 | 22,46 | 4,56 | 4,15 | 4,83 | 35,29 | 34,32 | 36,44 | 10,96 | 10,79 | 11,43 | 11,36 | 11,13 | 11,64 | 15,25 | 14,06 | 16,40 |
| Mato Grosso | 10,56 | 10,37 | 10,73 | 49,53 | 48,41 | 51,63 | 45,90 | 43,83 | 50,06 | 29,10 | 22,85 | 33,89 | 4,27 | 3,87 | 4,56 | 39,74 | 38,41 | 41,05 | 12,75 | 12,46 | 13,37 | 11,85 | 11,60 | 12,12 | 18,83 | 16,68 | 21,13 |
| Mato Grosso Sul | 10,84 | 10,78 | 10,91 | 46,49 | 45,50 | 48,58 | 45,98 | 43,83 | 50,16 | 28,63 | 23,29 | 33,01 | 5,23 | 4,87 | 5,38 | 40,10 | 38,85 | 41,41 | 16,45 | 16,09 | 16,90 | 11,55 | 11,28 | 11,87 | 19,75 | 18,24 | 21,45 |
| Minas Gerais | 11,15 | 10,98 | 11,28 | 44,85 | 43,80 | 46,89 | 45,84 | 43,78 | 50,12 | 23,91 | 18,91 | 28,13 | 4,06 | 3,78 | 4,27 | 41,06 | 39,42 | 43,10 | 14,82 | 14,05 | 15,19 | 12,76 | 12,07 | 13,07 | 20,64 | 18,82 | 22,91 |
| Pará | 9,66 | 9,57 | 9,72 | 39,03 | 38,40 | 40,37 | 45,83 | 43,68 | 49,98 | 22,78 | 17,80 | 27,47 | 3,91 | 3,55 | 4,20 | 37,56 | 36,56 | 38,85 | 11,66 | 11,43 | 12,20 | 12,40 | 12,21 | 12,64 | 15,68 | 14,59 | 16,80 |
| Paraíba | 9,52 | 9,36 | 9,59 | 37,52 | 36,93 | 38,80 | 45,94 | 43,85 | 50,17 | 21,79 | 16,75 | 26,07 | 4,62 | 4,09 | 4,96 | 37,22 | 35,99 | 38,40 | 13,72 | 13,40 | 14,30 | 15,69 | 15,06 | 15,94 | 17,03 | 15,43 | 18,71 |
| Paraná | 10,93 | 10,83 | 11,05 | 48,72 | 47,62 | 50,77 | 46,02 | 43,90 | 50,21 | 27,28 | 22,50 | 31,28 | 4,28 | 3,87 | 4,58 | 40,07 | 39,03 | 41,17 | 17,23 | 16,91 | 17,80 | 12,62 | 11,90 | 12,92 | 21,90 | 19,77 | 24,34 |
| Pernambuco | 10,30 | 10,13 | 10,42 | 39,09 | 38,43 | 40,62 | 45,86 | 43,80 | 50,06 | 22,70 | 18,00 | 26,87 | 4,62 | 4,26 | 4,88 | 38,10 | 37,03 | 39,27 | 14,03 | 13,72 | 14,68 | 12,73 | 12,53 | 13,00 | 18,61 | 17,05 | 20,42 |
| Piauí | 9,89 | 9,69 | 10,03 | 35,95 | 35,46 | 37,11 | 45,90 | 43,75 | 50,06 | 18,94 | 14,09 | 23,43 | 4,30 | 3,88 | 4,61 | 36,19 | 35,12 | 37,28 | 12,04 | 11,83 | 12,51 | 11,19 | 11,03 | 11,39 | 15,20 | 14,21 | 16,39 |
| Rio de Janeiro | 11,69 | 11,51 | 11,90 | 52,55 | 51,40 | 54,60 | 45,88 | 43,86 | 50,13 | 29,30 | 24,92 | 32,65 | 4,77 | 4,54 | 4,91 | 36,71 | 35,54 | 37,95 | 18,85 | 18,54 | 19,40 | 14,89 | 14,37 | 15,16 | 20,29 | 18,65 | 22,19 |
| Rio Grande do Norte | 10,41 | 10,23 | 10,54 | 38,88 | 38,16 | 40,41 | 45,87 | 43,77 | 50,11 | 24,90 | 19,37 | 29,45 | 4,51 | 4,04 | 4,83 | 38,27 | 36,93 | 39,55 | 13,19 | 12,90 | 13,62 | 10,93 | 10,61 | 11,26 | 16,40 | 15,20 | 17,69 |
| Rio Grande do Sul | 11,61 | 11,48 | 11,77 | 50,08 | 49,06 | 51,92 | 46,05 | 43,92 | 50,33 | 30,49 | 26,48 | 33,56 | 4,09 | 3,82 | 4,29 | 36,17 | 34,85 | 37,25 | 17,90 | 17,64 | 18,44 | 11,17 | 10,97 | 11,42 | 23,84 | 21,16 | 26,31 |
| Rondônia | 9,85 | 9,75 | 9,91 | 43,91 | 42,94 | 45,94 | 45,93 | 43,84 | 50,11 | 26,25 | 20,07 | 31,50 | 4,27 | 3,93 | 4,48 | 38,73 | 37,41 | 40,07 | 11,36 | 11,12 | 11,93 | 14,30 | 14,01 | 14,59 | 17,77 | 16,71 | 18,94 |
| Roraima | 9,51 | 9,37 | 9,62 | 44,38 | 43,70 | 45,59 | 45,87 | 43,74 | 50,08 | 25,80 | 20,46 | 30,21 | 4,33 | 4,03 | 4,57 | 38,01 | 37,03 | 39,13 | 10,84 | 10,68 | 11,31 | 13,14 | 12,93 | 13,38 | 18,03 | 16,67 | 19,67 |
| São Paulo | 11,34 | 11,19 | 11,51 | 54,56 | 53,40 | 56,51 | 45,66 | 43,51 | 49,85 | 29,21 | 24,69 | 33,00 | 4,41 | 4,21 | 4,61 | 37,81 | 36,76 | 39,01 | 16,69 | 16,44 | 17,26 | 11,37 | 11,11 | 11,75 | 22,07 | 19,51 | 24,76 |
| Santa Catarina | 12,19 | 12,03 | 12,31 | 51,59 | 50,44 | 53,66 | 45,98 | 43,82 | 50,20 | 27,46 | 22,40 | 31,98 | 3,89 | 3,60 | 4,10 | 40,04 | 39,09 | 41,08 | 24,93 | 22,03 | 26,24 | 12,83 | 12,57 | 13,11 | 20,54 | 17,80 | 23,34 |
| Sergipe | 10,52 | 10,35 | 10,63 | 40,97 | 40,30 | 42,27 | 45,95 | 43,79 | 50,12 | 23,64 | 18,17 | 28,07 | 4,61 | 4,20 | 4,99 | 38,24 | 37,04 | 39,39 | 15,96 | 15,60 | 16,65 | 11,34 | 11,10 | 11,60 | 16,38 | 15,11 | 17,85 |
| Tocantins | 10,05 | 9,76 | 10,19 | 41,05 | 40,29 | 42,50 | 45,89 | 43,75 | 50,05 | 22,11 | 15,56 | 27,61 | 4,19 | 3,72 | 4,59 | 37,45 | 35,86 | 39,00 | 9,51 | 9,24 | 10,01 | 11,84 | 11,51 | 12,10 | 17,05 | 16,04 | 18,31 |

**Supplementary Table 4.** Summary of boys and girls SEV Low birth weight and high body mass index from their specific time series from all 26 States.

|  | Newborn (1995 – 2017) | | | 10 to 14 y.o. (2005-2017) | | |
| --- | --- | --- | --- | --- | --- | --- |
|  | SEV Low birth weight | | | SEV High body mass index | | |
| *State* | Mean | min | máx | Mean | min | max |
| Acre | 7,38 | 7,07 | 7,86 | 21,55 | 16,92 | 25,23 |
| Alagoas | 7,57 | 7,36 | 7,94 | 19,79 | 15,94 | 22,91 |
| Amapá | 6,08 | 5,53 | 7,06 | 23,97 | 20,15 | 27,28 |
| Amazonas | 7,61 | 6,53 | 9,52 | 24,04 | 19,50 | 27,73 |
| Bahia | 7,81 | 7,11 | 9,01 | 22,72 | 18,30 | 26,20 |
| Ceará | 7,07 | 6,90 | 7,41 | 22,16 | 17,33 | 26,03 |
| Espírito Santo | 6,76 | 5,95 | 8,15 | 24,70 | 19,80 | 28,95 |
| Goiás | 7,56 | 7,46 | 7,75 | 27,10 | 20,87 | 31,93 |
| Maranhão | 6,64 | 6,15 | 7,34 | 15,16 | 11,72 | 18,11 |
| Mato Grosso | 7,06 | 6,39 | 8,16 | 29,23 | 22,75 | 33,78 |
| Mato Grosso Sul | 5,96 | 5,32 | 7,10 | 27,10 | 21,73 | 31,42 |
| Minas Gerais | 9,08 | 8,41 | 10,16 | 24,22 | 20,23 | 27,68 |
| Pará | 6,26 | 5,87 | 7,00 | 20,36 | 16,23 | 23,76 |
| Paraíba | 7,39 | 6,63 | 8,49 | 16,89 | 13,75 | 19,54 |
| Paraná | 7,59 | 7,15 | 8,31 | 28,38 | 23,58 | 32,02 |
| Pernambuco | 5,84 | 5,34 | 6,74 | 21,69 | 17,22 | 25,58 |
| Piauí | 5,72 | 5,26 | 6,47 | 18,75 | 14,91 | 21,88 |
| Rio de Janeiro | 8,61 | 7,95 | 9,70 | 27,10 | 23,50 | 30,24 |
| Rio Grande do Norte | 7,63 | 7,03 | 8,67 | 20,84 | 16,33 | 24,55 |
| Rio Grande do Sul | 9,34 | 9,11 | 9,66 | 31,58 | 26,91 | 35,19 |
| Rondônia | 6,01 | 5,57 | 6,71 | 23,04 | 17,88 | 27,41 |
| Roraima | 6,12 | 6,06 | 6,26 | 23,16 | 18,99 | 26,57 |
| São Paulo | 9,63 | 8,88 | 10,61 | 26,62 | 22,58 | 29,93 |
| Santa Catarina | 7,25 | 6,87 | 7,73 | 32,46 | 27,14 | 36,29 |
| Sergipe | 7,01 | 6,13 | 8,49 | 20,11 | 16,55 | 23,07 |
| Tocantins | 4,91 | 4,66 | 5,39 | 18,91 | 14,11 | 22,75 |
